# Regulatory effect of *Fructus Mume* decoction on gut microbiota in mice based on *16S rDNA* sequencing

**DOI:** 10.5455/javar.2026.m1034

**Published:** 2026-03-24

**Authors:** Jianhui Huang, Dongjie Huang, Chenfeng Ma, Peifu Wu

**Affiliations:** 1 College of Biology and Food Engineering, Southwest Forestry University, Bailongcun 300, Kunming, China

**Keywords:** *Fructus Mume*, gut microbiota, high-throughput sequencing, KM mice, molecular docking

## Abstract

**Objectives:** *Fructus Mume* has long been used to prevent diseases by modulating gut microbiota, enhancing intestinal barrier integrity, and boosting immune function. Therefore, it is necessary to clarify the specific connection between *Fructus Mume* and gut health.

**Materials and Methods:** This study employed *16S rDNA* sequencing to investigate the regulatory effects of *Fructus Mume* decoction on the gut microbiota structure in Kunming (KM) mice. 13-week-old male KM mice were randomly assigned to a control group and to low-, medium-, and high-dose *Fructus Mume* groups (*n* = 4 per group). Mice were administered distilled water or *Fructus Mume* decoction (0.42, 0.84, 1.68 gm/kg) via daily gavage (0.2 ml) for 7 consecutive days. Fecal samples were collected on days 1 and 7 post-withdrawal.

**Results:** At the phylum level, on day 1 post-withdrawal, the Bacteroidota abundance increased slightly in the high-dose group, while the Firmicutes_D abundance increased significantly. At the genus level, *CAG-873* and *Alloprevotella* exhibited significantly positive correlations with *Fructus Mume* dose, whereas *UMGS1994* and *Nanosyncoccus* showed significantly negative correlations (|*r*| > 0.4). Functional prediction indicated upregulation of carbohydrate metabolism and xenobiotic biodegradation and metabolism pathways in *Fructus Mume* groups. The latter pathway gradually downregulated post-treatment cessation. Molecular docking analysis revealed that kaempferol and quercetin in *Fructus Mume* decoction could interact with core proteins associated with intestinal diseases.

**Conclusions:** Gavage of *Fructus Mume* decoction at human-equivalent doses for one week altered intestinal flora composition in KM mice, reducing diversity and richness, and the dose of 0.84 gm/kg was more beneficial to gut health. These parameters gradually recovered post-withdrawal.

## 1. Introduction

*Fructus Mume*, which is the dried, nearly ripe fruit of *Prunus mume* from the Rosaceae family, is primarily found in regions south of the Yangtze River in China. This fruit contains a complex mix of bioactive compounds that support gut health through various mechanisms, such as reducing inflammation, enhancing immunity, shaping gut structure, regulating the microbiota, and even reversing drug resistance, as supported by studies [1, 2, 3, 4, 5, 6]. The gut microbiota plays a crucial role in animal health, productivity, and nutrient absorption, while intestinal health itself influences the composition of microbial communities. As a substance that serves both as food and medicine, *Fructus Mume* provides cost-effective, low-toxicity bacteriostatic effects and has been used for centuries to prevent disease [7]. It also helps strengthen intestinal barriers, though the specific ways in which its decoction alters gut microbiota are still not fully understood.

KM mice are especially valuable as research models because their gut microbiota composition, function, and immune response closely resemble those of humans [8]. The bioactivity of *Fructus Mume* stems from its rich array of phytochemicals, such as sterols and organic acids [9, 10]. Organic acids enhance gut motility and aid digestion, while flavonoids like kaempferol help regulate gut barriers and repair damage [11]. Vitamin C and polyphenols act as antioxidants, neutralizing harmful free radicals, and compounds such as tannic acid and anthocyanins may support gut mucosal health [12]. *In vivo* studies have confirmed these effects: Nie et al. [13] found that *Fructus Mume* pills increased beneficial probiotic populations in obese mice, while Zhang et al. [14] demonstrated that the remedy could reshape an imbalanced gut microbiota by reducing harmful pathogens and boosting *Lactobacillus* populations [5]. *Fructus Mume* also shows promise for targeting drug-resistant bacteria, as low concentrations have been shown to suppress ESBL-producing Enterobacterales and eliminate resistance plasmids, offering a potential solution for difficult-to-treat infections [15]. However, there are important caveats to consider. Prolonged exposure to high doses—such as 7.80 gm/kg/day for nine days—has been shown to reduce body weight and raise serum *TNF-α* [16] levels in mice, which could trigger chronic inflammation; this aligns with clinical reports of gastric discomfort following extended high-dose use. Thus, longer durations or higher doses of *Fructus Mume* decoction are not always beneficial. Another key question is whether its positive effects on gut microbiota persist after treatment stops.

While we have a partial understanding of how *Fructus Mume* affects the microbiota, most current research has focused on disease models, leaving unexplored the temporal changes in healthy animals and the direct interactions between microbes and the herb itself. In this study, KM mice were given different doses of *Fructus Mume* decoction through their stomachs for a week; high-throughput sequencing was then used to analyze the structure of their fecal microbiota to look at how different doses affected them and what changes occurred after the treatment was stopped, while molecular docking was employed to delve into the mechanisms behind these effects to help support the development of medicinal applications.

## 2. Materials and Methods

### 2.1. Experimental materials and ethical approval

Sixteen 13-week-old male KM mice were obtained from Henan Sikebeisi Biotech Co., Ltd. in China, which holds a production license (SCXK(Yu)2020-0005) and a qualification certificate (410000000000011406). All procedures involving animals were carried out in accordance with the requirements set by the Animal Welfare Ethics Committee of Southwest Forestry University, and the study received approval under the approval number SWFU-2023021. The standard irradiated diet, specifically mouse maintenance feed, was supplied by Jiangsu Collaborative Pharmaceutical Biotech Co., Ltd. in China, as detailed in Table 1. *Fructus Mume* was purchased from a licensed Chinese medicine dispensary, and after removing the pulp, the samples were ground into a fine powder, passed through a 40-mesh screen, and then stored in airtight containers.

**Table 1.** Nutritional composition specifications (as-fed basis).

Item	Value
Moisture, gm/kg	≤ 100
Crude Protein, gm/kg	≥ 180
Crude Fat, gm/kg	≥ 40
Crude Fibre, gm/kg	≤ 50
Crude Ash, gm/kg	≤ 80
Calcium (Ca), gm/kg	10–18
Total Phosphorus (P), gm/kg	6–12
Calcium to Phosphorus Ratio (Ca: P)	1.2:1–1.7:1

### 2.2. Preparation and administration of Fructus Mume decoction

*Fructus Mume* powder was dried at 45°C until a constant weight was reached. Following the decoction protocols outlined in the Management Specifications for Traditional Chinese Medicine Decoction Rooms in Medical Institutions, we began by soaking 100 gm samples in 400 ml of distilled water, maintaining a 1:4 weight-to-volume ratio, for 30 min. After soaking, the samples were decocted for an additional 30 min. The mixture was then filtered through filter paper, and the residue was subjected to a second decoction with 200 ml of distilled water, at a 1:2 weight-to-volume ratio, for 20 min, followed by another filtration. The combined filtrates were concentrated to a final volume of 200 ml, yielding a crude drug decoction at 0.5 gm/ml, which was then stored sealed at 4°C. To determine the appropriate mouse dosing, we referred to the maximum human dosage specified in the *Chinese Pharmacopoeia* [17], which is 12 gm/day. Using a human-mouse equivalent dose ratio of 9.1:1 based on body surface area and assuming a standard human weight of 65 kg, we calculated the maximum murine dose to be 1.68 gm/kg. We established four treatment groups for the experiment: a control group (CON) receiving 0.00 gm/kg, a low-dose group (L) receiving 0.42 gm/kg, a medium-dose group (Me) receiving 0.84 gm/kg, and a high-dose group (H) receiving 1.68 gm/kg, with each mouse receiving a daily gavage volume of 0.2 ml. The experiment took place at Southwest Forestry University’s Animal Breeding Base in October 2024. Four days before the trial began, the mice were randomly assigned to their respective groups (4 per group) and allowed to acclimatize. Daily gavage administration was carried out at 14:30 for seven consecutive days.

### 2.3. Fecal sample collection, DNA and RNA extraction, 16S rDNA sequencing, and qPCR

Fresh fecal samples were aseptically collected from individual mice at 08:30 on the first and seventh days after withdrawal. These samples were immediately placed on ice after collection and stored at –80°C to preserve their integrity. Total genomic DNA was extracted from the fecal microbiota using the E.Z.N.A.^TM^ MicroElute Genomic DNA Kit (Omega Bio-Tek, USA) according to the manufacturer‘s instructions. The quality and purity of the DNA were assessed using 1.0% agarose gel electrophoresis and NanoPhotometer^®^ spectrophotometry (IMPLEN, California, USA), respectively. The V3-V4 hypervariable regions of the *16S rDNA* were amplified by PCR, following the method described by Chen et al. [18]. The amplified products were then purified using the E.Z.N.A.^®^ Gel Extraction Kit from Omega Bio-Tek, USA, and quantified with a Qubit^®^ 4.0 Fluorometer from Thermo Fisher Scientific in Waltham, Massachusetts, USA. Sequencing libraries were prepared using the VAHTS^TM^ Universal Pro DNA Library Prep Kit for Illumina^®^ (Vazyme Biotech, Nanjing, China) and sequenced on the Illumina NovaSeq platform. The raw sequences were processed in QIIME2 (v2024.5) using the DADA2 pipeline for quality filtering, denoising, merging paired-end reads, inferring Amplicon Sequence Variants (ASVs), and removing singleton ASVs. All samples were rarefied to 47,286 sequences to ensure uniformity. Subsequent analyses in QIIME2 included taxonomic annotation, alpha diversity analysis, community composition analysis, differential abundance testing across taxonomic levels, and Principal Coordinate Analysis (PCoA) based on Bray-Curtis distances. Functional prediction was carried out using PICRUSt2 (v2.2.2-b) for KEGG annotation and COG classification.

Fecal samples collected from individual mice within the same group were combined and processed using the E.Z.N.A.^®^ Soil RNA Midi Kit from Omega Bio-Tek in the USA to extract total RNA. Next, cDNA libraries were prepared using the Evo M-MLV RT Mix Kit (Accurate Biotechnology, Changsha, China). For the quantitative analysis, the *gyrB* gene, which encodes DNA gyrase subunit B, was chosen as the reference gene, with the primers F: 5′-ACC CGG ACA AAC TGC GTT AT-3′ and R: 5′-GAT CCA TCG CTT CGT CGT CT-3′. The target genes under investigation were pta, which stands for phosphate acetyltransferase, with primers F: 5′-CTG GTT GAA GGT CTG GTC CC-3′ and R: 5′-ATA ACG CCG GTG ATG TTG GT-3′, and buk, or butyrate kinase, for which the primers were sourced from Jia et al. [19]. Quantitative real-time PCR (qPCR) was carried out on a CFX96^TM^ Real-Time System manufactured by Bio-Rad Laboratories in Hercules, CA, USA, utilizing the 2X Plus qPCR MasterMix from Biologyark in Shanghai, China. The qPCR protocol consisted of an initial hot-start activation at 95°C for 30 sec, followed by 40 cycles of denaturation at 95°C for 5 sec, annealing at 60°C for 30 sec, and extension at 72°C for 30 sec, with fluorescence acquisition at 72°C. Finally, a melt curve analysis was performed over the range of 60°C to 95°C.

### 2.4. Pharmacological profiling of bioactive compounds in Fructus Mume decoction

The target proteins were obtained from the PDB database, and then a protein-protein interaction (PPI) network was built using the STRING database to pinpoint the core targets. From the TCMSP database, active compounds in *Fructus Mume* decoction that interact with intestinal disease-related core proteins were selected based on pharmacokinetic criteria, specifically calculated octanol–water partition coefficient (AlogP) ≤ 5, oral bioavailability (OB) ≥ 30%, and drug likeness (DL) ≥ 0.18. Additional compounds that interact with core proteins involved in bacterial pathways, such as xenobiotic biodegradation and metabolism, carbohydrate metabolism, and cytoskeletal functional category proteins, were also screened using modified criteria, namely AlogP ≤ 5 and OB ≤ 10%. After that, molecular docking validation was performed using AutoDockTools 1.5.7.

### 2.5. Statistical analysis

The experimental data are shown as the mean ± standard deviation (SD). Statistical analyses and data visualization were performed using R version 4.4.3. The abundance data were normalized using Z-score normalization. To evaluate differences between groups, the Kruskal-Wallis test was applied, followed by post-hoc pairwise comparisons with adjusted p-values. For the association analysis, Spearman’s rank correlation coefficient was calculated. In this study, statistical significance was defined as an adjusted *p*-value less than 0.05.

## 3. Results

### 3.1. Alpha diversity of murine fecal microbiota

**Figure 1.**
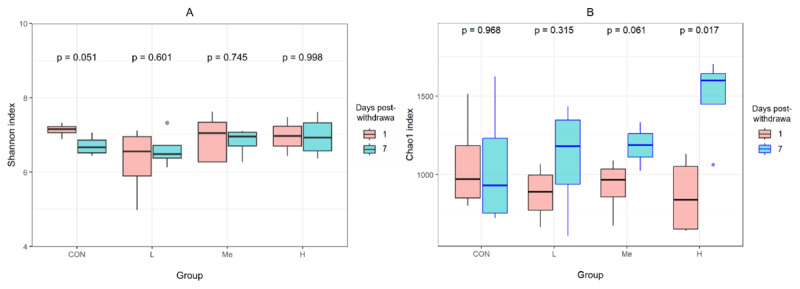
Temporal comparison of Shannon (A) and Chao1 (B) indices across experimental groups on 1 and 7 days post-withdrawal (the absence of annotation indicates no significant intergroup differences at either time point).

Alpha diversity analysis showed that when mice were given *Fructus Mume* decoction by gavage for 1 week, both diversity (measured by the Shannon index) and richness (measured by the Chao1 index) of their fecal microbiota decreased within 7 days after treatment. However, these measures gradually returned to normal after the treatment stopped. On the first day after stopping treatment, the Shannon index was higher in the control (CON) group than in any of the treatment groups. By the seventh day after stopping treatment, the medium-dose (Me) and high-dose (H) groups had higher Shannon indices compared to the other groups. When looking at changes over time, there was a near-significant (*p* = 0.051) drop in the Shannon index for the CON group between days one and seven, while the treatment groups maintained stable diversity levels, suggesting that their microbiota remained stable after treatment withdrawal, as shown in Figure 1A.

When examining community richness, as measured by the Chao1 index, the CON group had a value of 1064.03 ± 305.30, which was higher than all treatment groups on day 1. The treatment groups had the following values: L at 878.4 ± 179.6, Me at 925.40 ± 188.40, and H at 863.10 ± 219.94, with the H group showing the lowest value. By day 7 after withdrawal, the H group’s richness had risen to 1490.55 ± 291.79, nearing a significantly higher level compared to the CON group, which was at 1052.88 ± 424.61, with a *p*-value of 0.095 and a mean difference of 437.67. However, the differences between the other groups were not significant (*p*-values > 0.05). At this time point, a dose-dependent increase in the Chao1 index was observed. Over time, the H group’s richness increased significantly from day 1 to day 7, while the other groups showed increases that were not statistically significant, with *p*-values greater than 0.05 (Figure 1B).

### 3.2. Fecal microbiota structural analysis in mice

#### 3.2.1. Analysis of ASVs in mouse fecal flora

**Figure 2.**
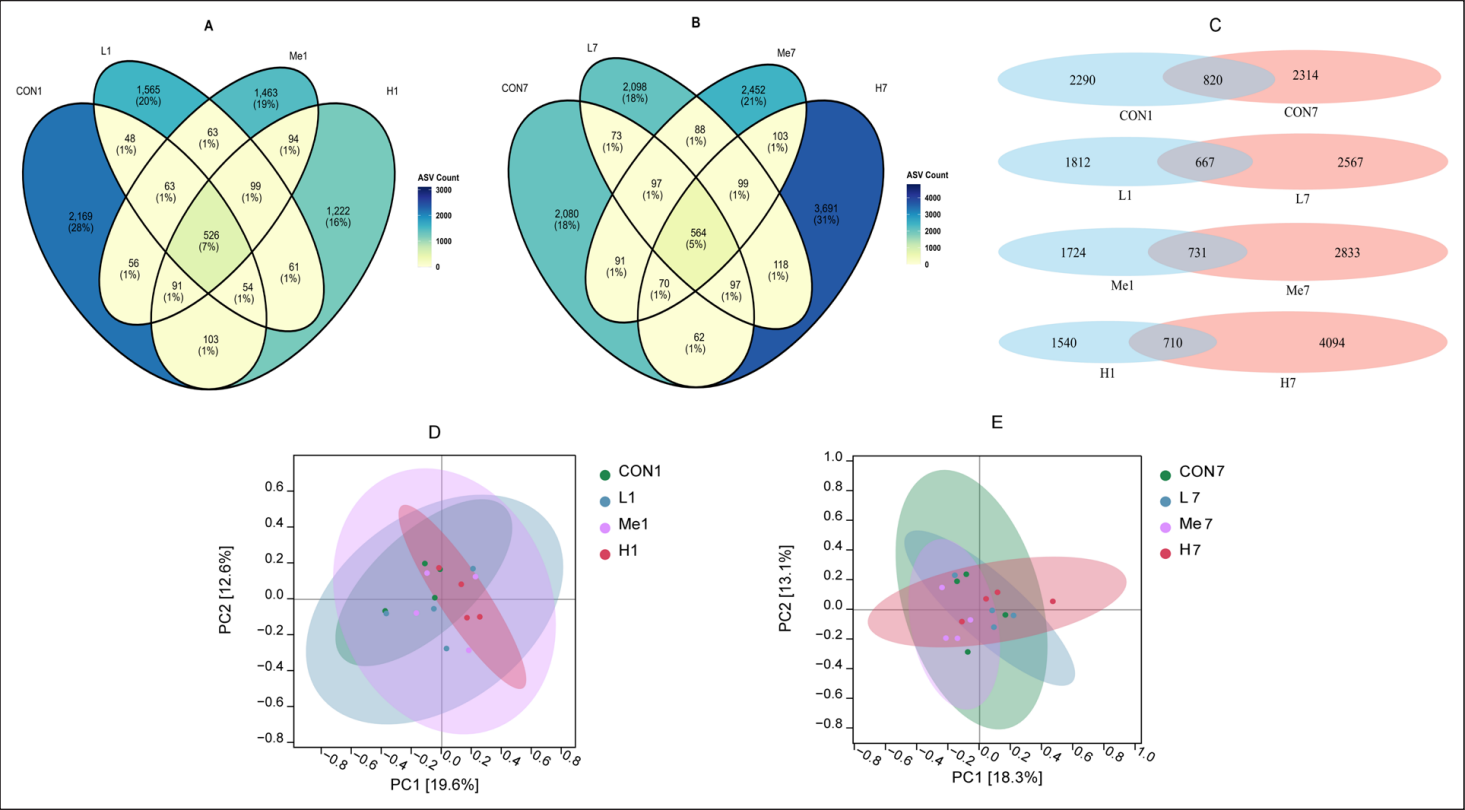
Venn diagrams of unique/shared ASVs in fecal samples on day 1 (A) and day 7 (B) post-withdrawal; longitudinal ASV changes between time points (C); PCoA ordination based on Bray-Curtis dissimilarity on day 1 (D) and day 7 (E) post-withdrawal.

ASV analysis showed that when mice were given a high dose of *Fructus Mume* decoction, their fecal microbiota composition changed during the active treatment phase, but these changes became less pronounced after the treatment stopped. One day after the treatment was withdrawn, the four treatment groups shared 526 ASVs, with the number of unique ASVs being as follows: the control group (CON) had 2169, the low-dose group (L) had 1565, the medium-dose group (Me) had 1463, and the high-dose group (H) had 1222, as shown in Figure 2A. This suggests that treatment with *Fructus Mume* reduced microbial richness. By day 7 after withdrawal, the number of unique ASVs in the treatment groups had increased significantly, with the control group having 2080, the low-dose group 2098, the medium-dose group 2452, and the high-dose group 3691, as seen in Figure 2B. A temporal analysis, shown in Figure 2C, revealed that the control group’s unique ASVs changed very little over time, whereas all treatment groups showed a notable recovery in richness, consistent with the findings on alpha diversity. PCoA based on ASV abundance indicated that on day 1 after withdrawal, the high-dose group’s microbiota composition was distinctly different from the others, as shown in Figure 2D, with the greatest dissimilarity between the control and high-dose groups (R = 0.23, *p* = 0.14). By day 7 after withdrawal, the largest difference was between the low-dose and medium-dose groups (R = 0.33, *p* = 0.06), as depicted in Figure 2E, suggesting that the treatment groups became more distinct from each other during the recovery period.

#### 3.2.2. Analysis of mouse fecal flora species composition and intergroup differences

**Figure 3.**
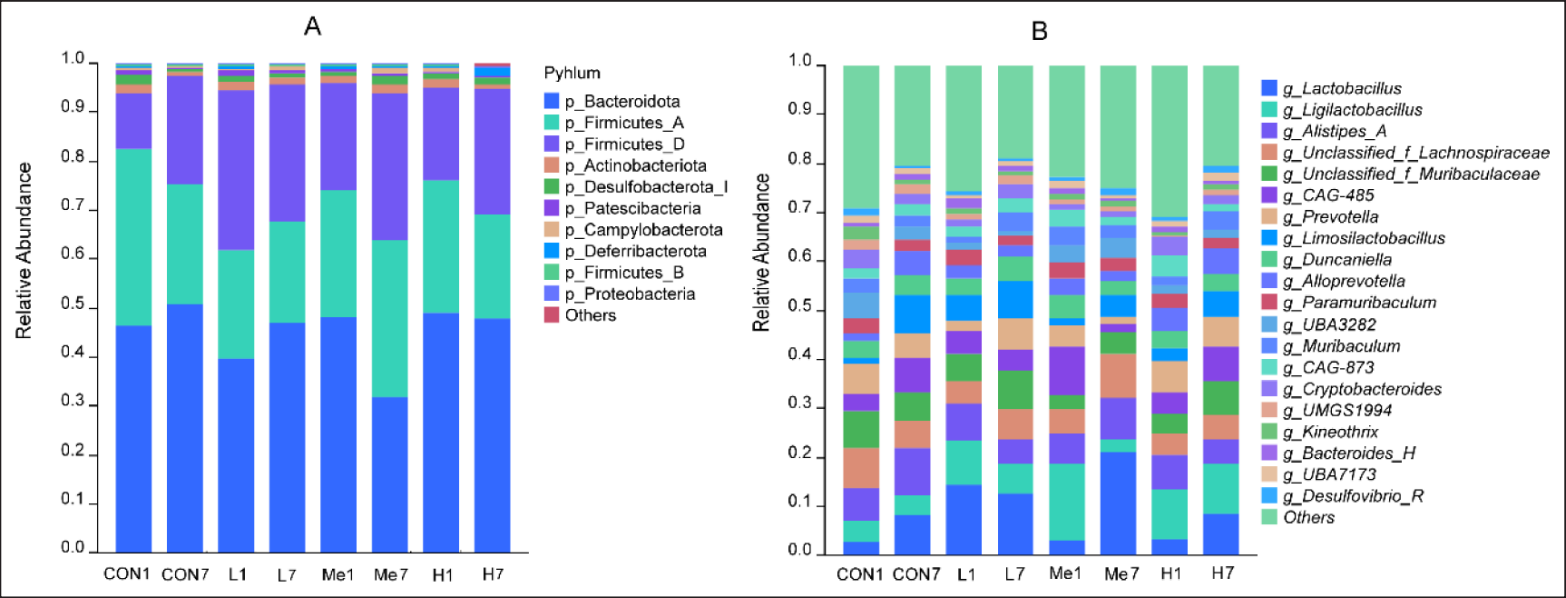
Microbial community composition at the phylum (A) and genus (B) levels across groups on days 1 and 7 post-withdrawal.

When we analyzed the composition of mouse fecal microbiota, we found that administering *Fructus Mume* decoction by oral gavage markedly altered the relative abundance of Firmicutes_D. This treatment also affected how the intestinal microbiota composition changed over time. Across all samples, we consistently detected nine bacterial phyla and 59 bacterial genera. Looking at the phylum level, as shown in Figure 3A, the main phyla in all groups were Bacteroidota, which made up 45.1% ± 6.2%; Firmicutes_A at 26.2% ± 5.4%; and Firmicutes_D at 23.9% ± 6.8%. The next most common phyla were Actinobacteriota, accounting for 1.4% ± 0.4%, and Desulfobacterota_I at 1.2% ± 0.4%. On the first day after stopping the treatment, the H group showed a slight increase in Bacteroidota compared to the CON group, with a log2 fold-change of 0.08, though this wasn’t statistically significant, but there was a notable rise in Firmicutes_D, with a log2 fold-change of 0.73, indicating possible changes in metabolism. By day seven after withdrawal, the Me group had a decrease in Bacteroidota, with a log2 fold-change of –0.20, which wasn’t significant, while Firmicutes_A and Firmicutes_D both increased, with log2 fold-changes of 0.37 and 0.47, respectively, also not significant compared to the CON group. When comparing the changes from day one to day seven, Bacteroidota dropped significantly in the Me group, with a log2 fold-change of –0.60, but stayed about the same in the H group, with a log2 fold-change of –0.03, which wasn’t significant.

In our genus-level analysis focusing on the top 20 genera as shown in Figure 3B, we found that *Fructus Mume* decoction had a notable impact on the relative abundances of several genera, including *Ligilactobacillus, UBA3282, Alloprevotella, CAG-873, Kineothrix*, and *UMGS1994*, while also influencing the overall dynamics of the microbiota. Just one day after the treatment was withdrawn, we observed significant changes in the abundances of *UBA3282, CAG-873, Kineothrix*, and *UMGS1994* in Group H compared to the CON group, with log2 fold-changes of –1.58, 1.11, –1.73, and –3.42, respectively. The change in *Alloprevotella* abundance in Group H was close to being statistically significant, showing a log2 fold-change of 1.58 with a *p*-value of 0.053. In GroupMe, *Ligilactobacillus, Alloprevotella*, Cryptobacteroides, and *UMGS1994* experienced substantial fold-changes relative to the CON group, with log2 fold-changes of 1.82, 1.22, –1.74, and –1.31, respectively, though none of these changes reached statistical significance, as all *p*-values were above 0.05. Seven days after stopping the treatment, the relative abundance of *Ligilactobacillus* in the high-dose (H) group was higher than in the control (CON) group, with the difference nearing statistical significance, showing a log2 fold-change of 1.30 and a *p*-value of 0.057. In contrast, the medium-dose (Me) group saw a significant rise in *Lactobacillus* levels, with a log2 fold-change of 1.36. These findings suggest a dose-specific pattern of proliferation, where *Ligilactobacillus* does well under high-dose conditions, while *Lactobacillus* benefits more from a medium dose. When we looked at the changes from day 1 to day 7 after withdrawal, we noticed a sharp increase in *Lactobacillus* levels in both the Me and H groups, with log₂ fold-changes of 2.76 (*p* = 0.029) and 1.35 (*p* = 0.073), respectively, while other groups showed only mild increases. This trend indicates that a medium-dose intervention might create a more favorable environment for *Lactobacillus* to multiply. Additionally, *Limosilactobacillus* saw a significant boost in the Me group, with a log2 fold-change of 1.66, and *CAG-873* experienced a sharp decline in the H group, with a log2 fold-change of –1.61.

#### 3.2.3. Analysis of differentially abundant taxa in mouse fecal microbiota

**Figure 4.**
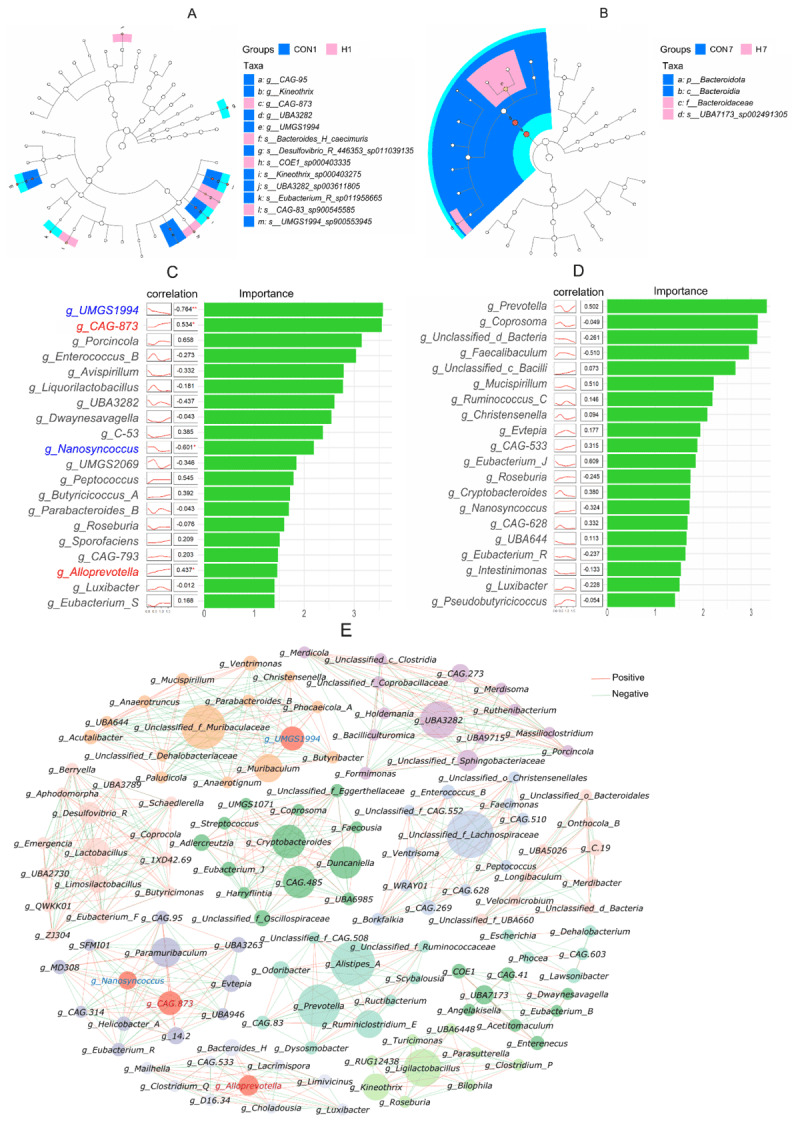
LEfSe analysis results on day 1 (A) and day 7 (B) post-withdrawal; correlation and random forest analysis results on day 1 (C) and day 7 (D) post-withdrawal (An asterisk (*) following a data point denotes a significant correlation (*p* < 0.05), while two asterisks (**) indicate a highly significant correlation (*p* < 0.01).); microbial interaction network (I).

After removing taxa with a relative abundance of less than 0.05%, we used Linear Discriminant Analysis Effect Size (LEfSe) analysis, with an LDA score threshold of 2, to identify biomarkers that differed between groups. Significant differences in microbial composition were found between the H and CON groups at both time points. On day 1 after withdrawal, genus-level biomarkers such as *CAG-95, Kineothrix, CAG-873, UBA3282*, and *UMGS1994* distinguished the H group from the CON group, as shown in Figure 4A. By day 7 after withdrawal, the CON group was characterized by biomarkers at the phylum level, specifically Bacteroidota, and at the class level, Bacteroidia, while the H group showed family-level biomarkers, specifically Bacteroidaceae, as seen in Figure 4B. No significant biomarkers were found in the other groups.

When we conducted a genus-level signature species analysis, we found some interesting dose-responsive taxa. On the first day after withdrawal, we noticed that *UMGS1994* and *Nanosyncoccus* decreased significantly in a way that depended on the dose they were exposed to. At the same time, *CAG-873* and *Alloprevotella* increased, as shown in Figure 4C, which hints that these changes might play a role in the therapeutic response. When we looked at the differences between groups on the seventh day after withdrawal, we saw that they didn’t have much to do with the treatment, as shown in Figure 4D. This suggests that the effects of the intervention were fading after the treatment stopped.

To explore how the four genera linked to different doses of *Fructus Mume* decoction affect intestinal microecology, we carried out a genus-level co-occurrence analysis of the gut microbiota, as shown in Figure 4E, where |*r*| > 0.7. The findings showed that *UMGS1994, Nanosyncoccus, CAG-873*, and *Alloprevotella*, with degrees of 14, 12, 12, and 9, respectively, each had significant correlations with multiple other genera, indicating their important roles in mediating how *Fructus Mume* decoction regulates the gut microbiota. The intestinal functions of *UMGS1994* and *Nanosyncoccus* are still not fully understood. Among the functionally characterized genera that are significantly positively correlated with both *UMGS1994* and *Nanosyncoccus*, all are beneficial bacteria, such as *Acutalibacter, Anaerotruncus*, and *Eubacterium_R*. On the other hand, the only functionally characterized genera that are significantly negatively correlated with them are *Christensenella* and *Butyribacter*. This suggests that *Fructus Mume* may promote beneficial bacteria by inhibiting the proliferation of *UMGS1994* and *Nanosyncoccus. CAG-873* and *Alloprevotella*, both of which produce short-chain fatty acids (SCFAs), show significant positive correlations with the functionally characterized genus *Bacteroides_H*, which also synthesizes SCFAs. However, the functions of genera that are significantly negatively correlated with *CAG-873* and *Alloprevotella* remain unclear. This indicates that *Fructus Mume* aqueous decoction may influence other bacteria by increasing the relative abundance of the *CAG-873* strain and the *Alloprevotella* genus.

### 3.3. Functional prediction analysis of murine fecal microbiota

#### 3.3.1. Results of KEGG pathway analysis and qPCR validation

**Figure 5.**
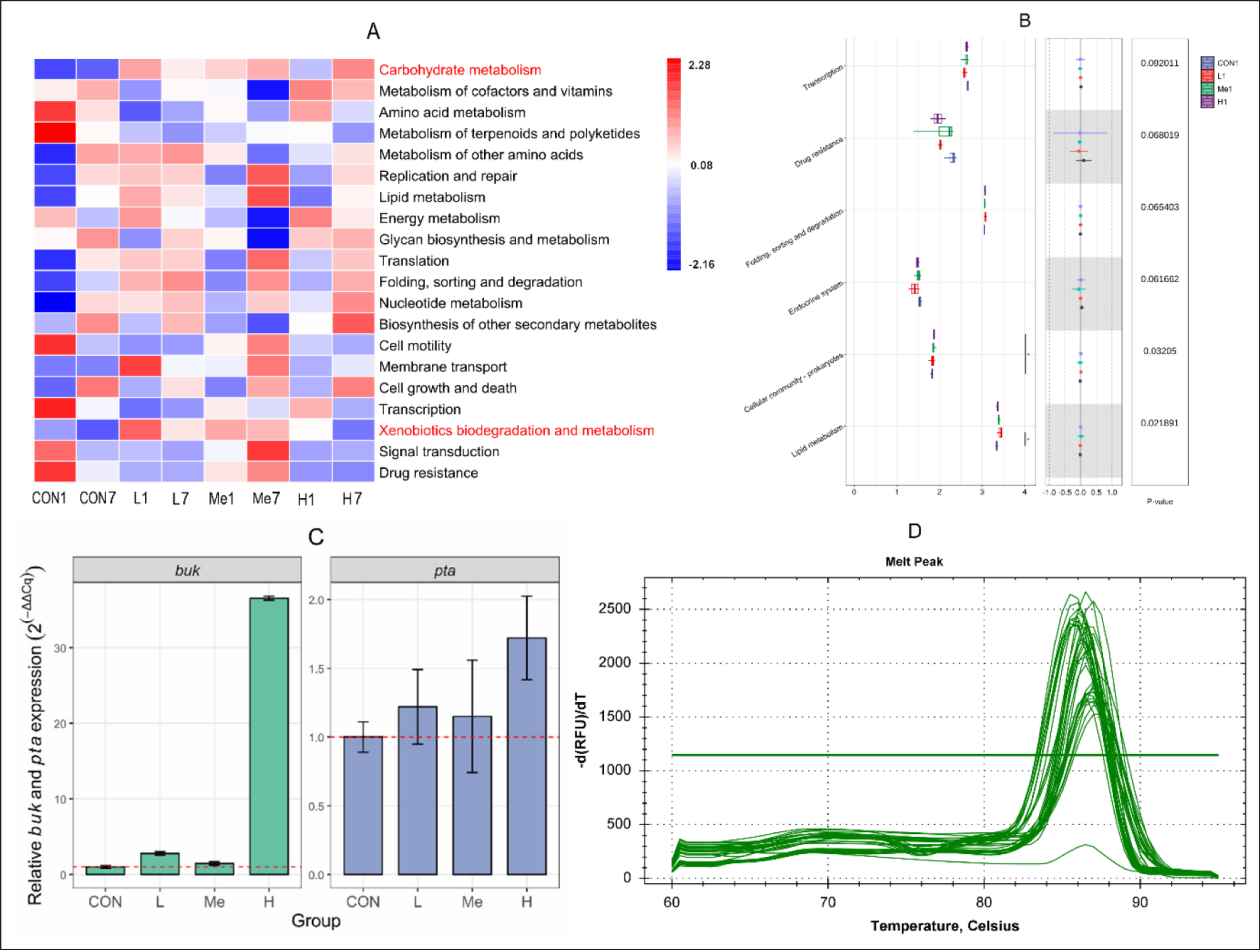
KEGG pathway analysis of fecal microbiota on day 1 and day 7 post-withdrawal (A) (In the heatmap, pathways positioned lower on the vertical axis indicate higher relative abundance, and color intensity reflects the pathway regulation status: blue hues denote downregulation, while red hues indicate upregulation in the respective treatment groups); differential pathway analysis on day 1 post-withdrawal (B); *pta* and *buk* gene expression in each group on day 1 post-withdrawal (C); and qPCR melting curve peaks (D). Error bars in (C) indicate the standard deviation of Cq values among technical replicates.

We picked this specific time for our analysis because earlier studies revealed that the gut microbiota composition undergoes a notable shift just one day after discontinuing treatment with *Fructus Mume* decoction. To delve deeper into how *Fructus Mume* affects the intestinal flora’s functional profile, we employed PICRUSt2 for KEGG pathway analysis. Our findings indicated that *Fructus Mume* enhances the carbohydrate metabolism as well as the xenobiotic biodegradation and metabolism pathways within the intestinal flora, as illustrated in Figure 5A. However, once the treatment was halted, the activity of this xenobiotic’s pathway gradually diminished.

One day after the treatment ended, carbohydrate metabolism proved to be far more active in the groups treated with *Fructus Mume* compared to the control group. When bacteria face xenobiotic stress, they adaptively reshape their carbohydrate metabolism to meet energy requirements, deal with toxic pressure, and manage the dynamics of metabolic intermediates, which is consistent with earlier research [16]. Initially, the pathway for xenobiotic biodegradation and metabolism was significantly more active in the treatment groups, but as time passed, its activity declined across all groups, as shown in Figure 5B. This suggests that the components in *Fructus Mume* decoction trigger xenobiotic stress in the intestinal flora, which then boosts the bacteria’s ability to metabolize xenobiotics. However, it’s important to note that the functional predictions in this study are based on *16S rDNA* sequencing data, which isn’t entirely accurate. That’s why we conducted preliminary qPCR tests to provide additional support for these predictions.

*Buk* and *pta* are crucial for bacterial carbon and energy metabolism, as noted in reference [20], which is why we decided to use a straightforward qPCR test to examine the expression levels of *buk* and *pta* in pooled fecal samples from each group of mice, aiming to confirm our hypotheses. Upon comparing these groups to the control group, we observed that the H, Me, and L groups exhibited significantly higher *buk* expression, with increases of 3555%, 45%, and 178%, respectively; for *pta*, the corresponding increases were 72%, 15%, and 22%, as shown in Figures 5C and 5D. These findings align precisely with what we had predicted.

#### 3.3.2. COG functional annotation analysis

**Figure 6.**
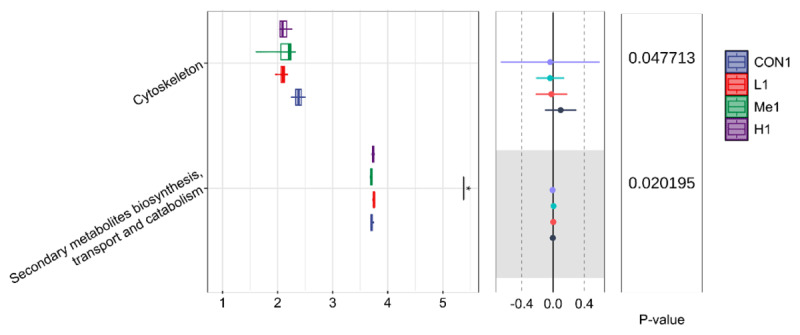
Differentially abundant COG functional categories across groups on day 1 post-withdrawal.

The COG functional annotation, which was performed using PICRUSt2, showed strong alignment with the findings from the KEGG pathway analysis. On day 1 after withdrawal, the main functional categories were similar across all groups, but when we examined the less abundant categories more carefully, we noticed a significant rise in cytoskeleton-related annotations in the H group compared to the others, as shown in Figure 6. This increase could suggest underlying processes related to the recovery of the intestinal microbiota.

### 3.4. Molecular docking analysis of Fructus Mume compounds with mouse host proteins and bacterial targets

**Figure 7.**
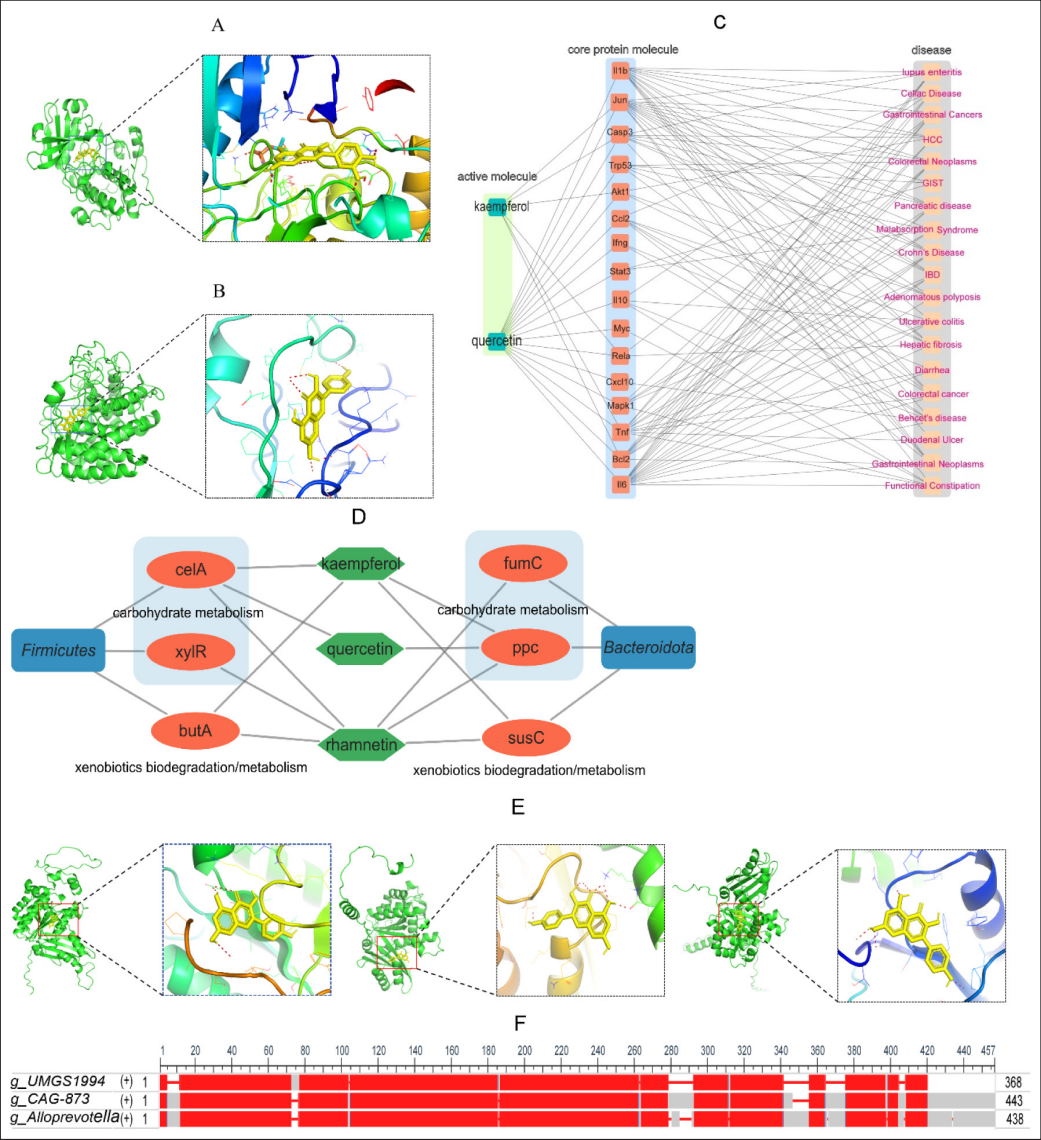
Quercetin-Akt1 (A) complex; kaempferol-Firmicutes celA (B) interaction; regulatory network of kaempferol/quercetin with intestinal disease-related core proteins (C); regulatory network of kaempferol/ /quercetin/rhamnetin with bacterial target proteins (D); differential binding sites of kaempferol on genus-specific FtsZ proteins (E); multiple sequence alignment of FtsZ proteins across bacterial genera (F).

To better understand how *Fructus Mume* decoction affects the intestinal flora in mice, we used molecular docking techniques, approaching the problem from two angles: one focused on how the decoction’s active compounds might interact with the host intestine and the other examining their direct interactions with gut bacteria. What we found was a dual mechanism of action. The decoction seems to influence the gut environment partly by strengthening intestinal immunity, which is an indirect approach, while at the same time, its molecular components directly bind to bacterial proteins. These interactions have an impact on several key microbial pathways, including carbohydrate metabolism, xenobiotic degradation, and cytoskeletal dynamics, ultimately reshaping the microbial community’s structure. When we looked at how the decoction affects the mouse intestinal tract, molecular docking analysis showed that kaempferol and quercetin—two active compounds identified *in silico* from the TCMSP database—can bind to multiple important proteins linked to various intestinal diseases (Table 2 and Figure 7A, C). This binding may enhance intestinal immune function, which then indirectly influences the makeup of the gut microbiota.

**Table 2.** Docking energies of active molecules against mouse-derived proteins.

Core Target Proteins of Intestinal-related Diseases	Molecular Docking Energy (kcal/mol)	
	Quercetin	Kaempferol
Il1b	–7.49	—
Il6	–7.46	—
Jun	–7.42	–7.68
Tnf	–7.50	–8.07
Trp53	–7.34	—
Bcl2	–6.66	–6.63
Casp3	–6.57	–7.06
Akt1	–8.57	–7.78
Ccl2	–6.63	—
Rela	–9.36	–9.77
Stat3	–6.99	—
Myc	–6.31	—
Mapk1	–8.09	—
Il10	–6.80	—
Ifng	–6.10	—
Cxcl10	–6.75	—

When we look at how the decoction directly affects intestinal bacteria, we used *in silico* methods to screen four active molecules—afzelin, thujin, rhamnetin, and amygdalin—from the TCMSP database. Take afzelin and thujin, for example; they don’t cross bacterial cell membranes very well, but gut bacteria secrete glycosidases that can break them down into kaempferol and quercetin, respectively, and these two compounds are much better at getting through cell membranes [21]. Amygdalin, however, is broken down by gut β-glucosidase into benzaldehyde and HCN, which then influence bacterial survival and metabolism by changing the availability of carbon sources, driving antibacterial competition, and prompting bacteria to adapt to toxicity [22]. Rhamnetin is unique because it can enter bacterial cells without needing to be modified first. Molecular docking analysis revealed that kaempferol, quercetin, and rhamnetin can bind to certain core proteins involved in xenobiotic biodegradation/metabolism and carbohydrate metabolism pathways in Firmicutes and Bacteroidota, which ultimately affects the composition of the microbiota (Table 3 and Figure 7B, D). Specifically, kaempferol had a strong binding affinity to the butA, celA, susC, and ppc proteins; quercetin bound well to celA and ppc, and rhamnetin showed good binding to butA, celA, xylR, susC, fumC, and ppc.

**Table 3.** Docking energies of active molecules against bacterial proteins.

Phylum	Target protein	Molecular Docking Energy (kcal/mol)		
		Kaempferol	Quercetin	Rhamnetin
*Firmicutes*	sepF	–6.29	–6.13	–6.42
	walR	–6.22	–6.49	–6.45
	butA	–7.07	–6.65	–7.17
	celA	–7.91	–7.45	–7.92
	xylR	–5.90	–5.69	–7.01
*Bacteroidota*	dcrH	–6.64	–6.07	–6.54
	susC	–7.01	–6.91	–8.02
	fumC	–6.38	–5.81	–7.65
	ppc	–7.33	–7.55	–7.83
	porA	–5.92	–6.20	–6.13

Characteristic genus analysis showed that when we upped the dose of *Fructus Mume*, the relative amounts of *UMGS1994* and *Nanosyncoccus* dropped significantly, while *CAG-873* and *Alloprevotella* saw a notable increase. At the same time, COG functional prediction pointed out that the high-dose group had a bigger share of cytoskeleton-related categories. Considering that only *UMGS1994, CAG-873*, and *Alloprevotella* are marked with the FtsZ protein—a crucial protein for regulating the cytoskeleton—in the NCBI database, we picked FtsZ to do molecular docking with kaempferol. This was done to obtain a preliminary view of the functional differences within the COG cytoskeleton category. The outcomes revealed differences in the FtsZ protein sequences among these three genera, as shown in Figure 7E. Molecular docking analysis further uncovered that the absolute docking energy value between kaempferol and the FtsZ protein of *UMGS1994* was greater than that for *CAG-873* and *Alloprevotella*, as detailed in Table 4 and Figure 7F. It’s worth mentioning that *UMGS1994* is part of the Firmicutes group, while the other two genera fall under Bacteroidota. These findings imply that the active ingredients in *Fructus Mume* have varying binding preferences for core proteins across different bacterial species, likely due to differences in protein sequences, which in turn could lead to structural shifts in the gut microbiota.

**Table 4.** Docking energies of kaempferol against FtsZ across bacterial genera.

Genus	Molecular Docking Energy (kcal/mol)
**Kaempferol**
*UMGS1994*	–7.31
*CAG-873*	–6.19
*Alloprevotella*	–5.60

## 4. Discussion

For this study, we examined the composition of the fecal microbiota in mice given different doses of *Fructus Mume* decoction by gavage. We found that Bacteroidota, Firmicutes_A, and Firmicutes_D were the main phyla present, which aligns with Huang et al. [23], who found the same phyla in the intestinal microbiota of six common experimental rodent strains. To give some context, Bacteroidota is dominant in the mammalian gut because it has co-evolved with the host over a long time [24]. Current research shows that apolipoproteins from intestinal epithelial cells in both mice and humans can specifically recognize and attach to ceramide-1-phosphate on the membranes of Bacteroidales. At the same time, outer membrane vesicles from Bacteroidales activate dendritic cells and commensal-specific T lymphocytes in the nearby intestinal area [25].

Seven days after withdrawal, the high-dose group showed the highest relative abundances of Bacteroidota and Firmicutes_D, indicating that *Fructus Mume* decoction, when administered at specific doses, can disrupt the balance of the intestinal microbiota in mice, potentially affecting immune function and the absorption and utilization of nutrients. Upon examining temporal changes, we observed that the microbiota composition shifted dynamically, with differences between groups gradually diminishing over time.

At the genus level, *Ligilactobacillus*, already the dominant in the mouse intestinal microbiota, showed a significant increase in relative abundance in the medium-dose group. Most species within this genus play crucial roles in enhancing the intestinal barrier, preventing pathogens from taking hold, regulating immune responses, and improving nutrient absorption, as noted in reference [26]. The inconsistent fluctuations in their levels across treatment groups suggest that *Fructus Mume* may not have a direct impact on these taxa. This observation leads to the conclusion that the 0.84 gm/kg dose—one of the three doses tested—may provide greater benefits for gut health. Additionally, we discovered positive dose-dependent relationships between the decoction dose and the abundances of *CAG-873* and *Alloprevotella*, as well as negative correlations with *UMGS1994* and *Nanosyncoccus*. These uncultured genera might be the key players in how *Fructus Mume* interacts with the microbiome.

Our KEGG and COG analyses revealed that *Fructus Mume* decoction can regulate the function of the fecal microbiota by boosting pathways involved in carbohydrate metabolism and the biodegradation and metabolism of xenobiotics. Xu et al. [27] previously observed that *Fructus Mume* decoction can inhibit the biosynthesis of Streptococcus mutans, a finding that supports our functional results and highlights the decoction’s multi-target action through constituents that both weaken biosynthesis, such as organic acids [28], and directly interact with intracellular targets. Research in rats has demonstrated that most afzelin can reach the distal intestine [29], while thujin can enter the rectal lumen to act directly on bacteria and help enrich probiotics [30], confirming that both compounds can reach the end of the digestive tract. However, these compounds have low permeability through bacterial cell membranes. They can be broken down by glycosidases secreted by gut bacteria into kaempferol and quercetin, respectively—molecules that can more easily pass through bacterial membranes—which then directly interact with essential proteins involved in key bacterial metabolic pathways. Molecular docking studies showed that kaempferol has a higher binding energy (absolute value) with FtsZ proteins from *UMGS1994* compared to those from *CAG-873/Alloprevotella*. FtsZ is a central regulator of bacterial division and the cytoskeleton [31], and kaempferol—similar to quercetin, which is known for its broad-spectrum antibacterial activity, ability to enhance sensitivity, and diverse bacterial targeting [32]—may compete with GTP to bind FtsZ proteins, thereby preventing bacterial proliferation. This difference in binding affinity may explain the observed shifts in microbial community structure. While these functional insights and mechanistic hypotheses have received some initial support through qPCR validation, they are primarily based on PICRUSt2 analysis of *16S rRNA* gene data—a method that, by its nature, isn’t as precise as shotgun metagenomics, which directly identifies functional genes. Because of this limitation, we intend to conduct further studies using direct metagenomic sequencing and GC-MS to confirm and refine these findings, thereby improving the reliability of our functional conclusions.

The decline in fecal microbiota diversity and richness we observed following treatment corresponds with *Fructus Mume*’s established antibacterial properties. Interestingly, our molecular docking analysis revealed that two of its key active ingredients, kaempferol and quercetin, can bind to core proteins associated with various host intestinal diseases. Research has demonstrated that both compounds can block the NF-κB pathway and reduce levels of pro-inflammatory cytokines, as cited in references [33, 34]. Proteins such as Rela, Tnf, Il6, Il1b, Ccl2, and Cxcl10, which are listed in Table 2, are involved in the NF-κB pathway, whereas Il10, Trp53, Casp3, and Akt1 are strongly linked to pro-inflammatory reactions. Based on this, we hypothesize that *Fructus Mume*’s aqueous decoction enhances intestinal immunity, fostering an environment that’s unfavorable for opportunistic bacteria. This aligns with previous findings that antibiotics disrupt microbial balance, while *Fructus Mume* helps counteract the reduction in microbial richness caused by antibiotics, as noted in references [35, 36]. Therefore, the drop in diversity results from both increased immunity and direct regulation within the gut lumen. Meanwhile, the recovery of diversity and richness after treatment ends suggests that *Fructus Mume*’s effects are relatively short-lived.

We should also note the limitations of this study. For one thing, while our sample size—four mice per group—aligns with the standard for this kind of exploratory research, it might still yield inconsistent findings due to individual differences in intestinal microbiota, as evidenced by the marginal significance (*p* = 0.051). Future studies should use larger sample sizes to boost statistical power and improve reproducibility of results, thereby helping confirm and expand on these findings. Another limitation is that *Fructus Mume* decoction has a complex composition, and we don’t yet fully understand how its key components are distributed in the intestine—this requires further investigation using research into multi-component synergistic effects and LC-MS/MS tracking technology. Additionally, we still haven’t completely determined the exact mechanism by which it acts in living organisms. Continuing in-depth research into how *Fructus Mume* decoction regulates the microbiota will certainly support its development as a drug and its clinical applications.

## 5. Conclusions

When KM mice were given *Fructus Mume* decoction orally at a dosage of 1.68 gm/kg over the course of a week, significant changes were observed in the composition of their gut microbiota. There was a decrease in both diversity and richness of the gut microbes, although the lower dose of 0.84 gm/kg was found to be gentler on the gut, with its effects gradually diminishing after the treatment ended. The decoction also led to an increase in the levels of Bacteroidota and Firmicutes, which might have thrown off the balance and interactions among the microbial community. Interestingly, higher doses of the decoction caused a rise in *CAG-873* and *Alloprevotella*, while *UMGS1994* and *Nanosyncoccus* levels dropped. These results indicate that *Fructus Mume* decoction could potentially impact gut health not only by indirectly boosting the immune system but also through direct bioactive interactions with bacteria, providing valuable insights into its possible therapeutic uses.

## Data Availability

The data presented in this study are available from the corresponding author upon reasonable request.

## List of abbreviations

DNA, deoxyribonucleic acid; rDNA, ribosomal DNA; KM mice, Kunming mice; ESBL, extended-spectrum β-lactamase; qPCR, quantitative real-time PCR; TNF-α, tumor necrosis factor-alpha; ASV, amplicon sequence variant; PCoA, principal coordinate analysis; KEGG, Kyoto Encyclopedia of Genes and Genomes; COG, Clusters of Orthologous Groups of Proteins; PPI, Protein-Protein Interaction; AlogP, apparent log partition coefficient; OB, oral bioavailability; DL, drug-likeness; LEfSe, linear discriminant analysis effect size; SCFAs, short-chain fatty acids; GC-MS, gas chromatography-mass spectrometry; LC-MS/MS, liquid chromatography-tandem mass spectrometry.

## References

[1] Li JM, Feng SS, Liu X, Jia X, Qiao FL, Guo JL (2022). Effects of traditional Chinese medicine and their active ingredients on drug-resistant bacteria. Front Pharmacol.

[2] Yang X, Chu L (2024). Study on the effect of Wumei Pill on apoptosis of colon cells in rats with ulcerative colitis and its mechanism by regulating miR-146a. Chin J Immunol.

[3] Chen J, Zhou GZ (2020). Effect of modified Wumei Pill combined with azintamide and probiotics on stool property and intestinal mucosal barrier function for patients with diarrhea after LC. J Sichuan Tradit Chin Med.

[4] Zhao MJ, Zhao Q, Guan Z, Liu QW, Zhou HY, Huang QW (2023). Effect of *Panax ginseng* and *Fructus Mume* on intestinal barrier and gut microbiota in rats with diarrhea. J Med Food.

[5] Huang Y, Wu CX, Guo L, Zhang XX, Xia DZ (2022). Effects of polysaccharides-riched *Prunus mume* fruit juice concentrate on uric acid excretion and gut microbiota in mice with adenine-induced chronic kidney disease. Curr Res Food Sci.

[6] Zhang J (2021).

[7] Zhu JN, Fu CL, Li SL, Zhang XF (2024). Application progress and the study on the mechanism of Chinese Medicine additives against porcine reproductive and respiratory syndrome virus. Feed Ind.

[8] Round JL, Mazmanian SK (2009). The gut microbiota shapes intestinal immune responses during health and disease. Nat Rev Immunol.

[9] Zhang JC, Liang H, Wang Y, Xi W, Li ZG (2021). Research progress of pharmacological action of Wumei (*Fructus Mume*). J Liaoning Univ Tradit Chin Med.

[10] Wang Y, Zhao GY, Xue SP, Wu GT, Duan HJ, Ren Y (2023). Wumeiwan and its similar prescriptions in the treatment of gastrointestinal diseases. Pharmacol Clin Chin Mater Med.

[11] Zhang J, Li KL, Fu XL, Zhang YQ, Liang YH, Liu QH (2025). Modulation of intestinal mucosal barrier in animals by 2 plant flavonols. J Fujian Agric Forest Univ.

[12] Ren N, Han XZ, Liu WW, Li Y, Li SH, Shang HT (2025). Research progress on effective ingredients of Wumei Pill (Wumeiwan) and its single drug in digestive system diseases. Chin Arch Tradit Chin Med.

[13] Nie KX, Zhao Y, Su H, Xu LJ, Zou X, Wang KF (2021). Effect of Wu-Mei-Wan on gut microbiota in obese mice. Chin J Hosp Pharm.

[14] Zhang ZQ, Xi SM, Miao XY, Zhao S, Wang YJ, Zhao X (2024). Effects of Wumei Papaya decoction on the gut microbiota and enzyme activity in mice with dysbiosis. J Tianjin Univ Tradit Chin Med.

[15] Gui Y, Huang LH, Hu XY, Yan YZ (2013). Ten kinds of Traditional Chinese Medicine inhibit ESBL-producing *Escherichia coli in vitro*. Chin Mod Dr.

[16] Zhou L, Wu F, Ou P, Li H, Zhuang WQ (2025). Non-electroactive bacteria behave variously in AnMBR biofilm control using electric field. Water Res.

[17] Zhang J, Li Z, Zhang Y, Guo YL, Zhu YR, Xia WX (2023). Mume Fructus (*Prunus mume* Sieb. et Zucc.) extract accelerates colonic mucosal healing of mice with colitis induced by dextran sulfate sodium through potentiation of cPLA2-mediated lysophosphatidylcholine synthesis. Phytomedicine.

[18] Chen QX, Ma XM, Xing ZS, Zhao X, Zu H, Guo ZW (2023). Antibiotic conditioning shapes pseudosterile mouse models by deleting colonic microbes rather than small intestinal microbes. Microbiol Spectr.

[19] Jia X, Verbrugghe A, Lourenço M, Cools A, Liu DJX, Marzorati M (2017). The response of canine faecal microbiota to increased dietary protein is influenced by body condition. BMC Vet Res.

[20] Won HI, Watson SM, Ahn JS, Endres JL, Bayles KW, Sadykov MR (2021). Inactivation of the Pta-AckA pathway impairs fitness of *Bacillus anthracis* during overflow metabolism. J Bacteriol.

[21] Li QY, Gao B, Siqin B, Qian H, Zhang R, Meng XX (2021). Gut microbiota: A novel regulator of cardiovascular disease and key factor in the therapeutic effects of flavonoids. Front Pharmacol.

[22] Jaswal V, Palanivelu J, Ramalingam C (2018). Effects of the gut microbiota on Amygdalin and its use as an anti-cancer therapy: Substantial review on the key components involved in altering dose efficacy and toxicity. Biochem Biophys Rep.

[23] Huang SW, Min FG, Wang J, Luo YZ, He LF, Chen ML (2021). Comparative study of intestinal flora in common mice and rats. Acta Lab Anim Sci Sin.

[24] Yao WN, Yan S, Xue Y, Zhao YL, Su H, Song YL (2023). Research progress on fecal microbiota transplantation in mammals. Chin J Anim Sci Vet Med.

[25] Wei M, Hu ZY, Xu LL, Bian XY, Wu L, Yin SY (2022). Quercetin positively affects gene expression profiles and metabolic pathway of antibiotic-treated mouse gut microbiota. Front Microbiol.

[26] Yang Y, Song X, Wang GQ, Xia YJ, Xiong ZQ, Ai LZ (2024). Understanding *Ligilactobacillus salivarius* from probiotic properties to omics technology: A review. Foods.

[27] Xu JF, Dong YR, Xu WJ, Fang JH, Li SS, Ping YL (2020). Study on the *in vitro* inhibitory effect of *Fructus Mume* extract on *Streptococcus mutans* in the oral cavity. Zhejiang J Tradit Chin Med.

[28] Tao Y, Hu XH, Cao F, Yun FL, Jia KW, Zhang MX (2025). Targeting symbionts by apolipoprotein L proteins modulates gut immunity. Nature.

[29] Li MQ, Li WL, Dong YX, Zhan C, Tao TT, Kang MC (2025). Advances in metabolism pathways of theaflavins: Digestion, absorption, distribution and degradation. Crit Rev Food Sci Nutr.

[30] Zhu Y, Yuan J, Sun WB, Liu TT (2022). Research progress on pharmacological effects and clinical application of Wumei (*Fructus Mume*). J Liaoning Univ Tradit Chin Med.

[31] Fujita J, Kasai K, Hibino K, Kagoshima G, Kamimura N, Tobita S (2025). Structural basis for the interaction between the bacterial cell division proteins *FtsZ* and *ZapA*. Nat Commun.

[32] Periferakis A, Periferakis K, Badarau IA, Petran EM, Popa DC, Caruntu A (2022). Kaempferol: Antimicrobial properties, sources, clinical, and traditional applications. Int J Mol Sci.

[33] Alshehri AS, El-Kott AF, El-Kenawy AE, Zaki MSA, Morsy K, Ghanem RA (2022). The ameliorative effect of kaempferol against CdCl-mediated renal damage entails activation of Nrf2 and inhibition of NF-kB. Environ Sci Pollut Res Int.

[34] Aghababaei F, Hadidi M (2023). Recent advances in potential health benefits of quercetin. Pharmaceuticals.

[35] Li YF, Ma WT, Cao Y, Chen YG, Wu XH (2021). Ameliorating effect of *Fructus Mume* on dysbiosis of murine gut microbiota induced by antibiotics. Nat Prod Res Dev.

[36] Tang QM, Han X, Yang GY, Chen R, Wang WJ, Tu XH (2024). Macrogenomics-based study of the mechanism of GeGen QinLian decoction in ameliorating dysbiosis in a rat model of antibiotic-associated diarrhea. Acta Lab Anim Sci Sin.

